# Editorial: Bio-materials for Cardiovascular Diseases

**DOI:** 10.3389/fcvm.2021.670964

**Published:** 2021-03-31

**Authors:** Paola Campagnolo, Maurizio Pesce

**Affiliations:** ^1^Section of Cardiovascular Sciences, Faculty of Health and Medical Sciences, School of Biosciences and Medicine, University of Surrey, Guildford, United Kingdom; ^2^Unità di Ingegneria Tissutale Cardiovascolare, Istituto di Ricerca e Cura a Carattere Scientifico, Milan, Italy

**Keywords:** biomaterials, scaffolds, tissue engineering, bioimplant, cardiovascular

The supply of transplants or autologous materials for the repair or replacement of defective cardiovascular tissues is largely inadequate and it does not fulfill the increasing demand resulting from the generalized aging of the population. Although the mortality has decreased over time thanks to improvements in prevention and new intervention protocols, the quality of life of the patients is still often poor, due to the limited regenerative capacity of the cardiovascular tissues (e.g., the myocardium) or the use of implant devices made with non-living tissues not endowed with regeneration ability (e.g., the bioprosthetic valves). In the last decades, the growth of the tissue engineering as a novel interdisciplinary framework to supply living materials able to regenerate lost tissues has made huge steps in refinement. This progress has been promoted by advancements in fabrication and refinement of biomaterials, and the possibility to combine cells with permanent or bio-absorbable scaffolds able to provide the necessary geometrical, chemical, and mechanical information to instruct proper cellular behavior. The aim of the present Research Topic was to collect experimental work and review articles illustrating improvements in the state-of-the-art for the production and application of biomaterials for the preparation of cardiac, valve, and vascular tissue replacements.

In two contributions within the collection, the use of dynamic culture bioreactors for the repopulation of a cell-free matrix with cardiovascular cells was investigated with the aim of producing material for reconstructive surgery of congenital heart disease or valve replacement. In the study by Cathery et al., the Authors combined a commercial porcine-derived extracellular matrix patch with umbilical cord derived pericytes, to generate a vascular graft with increased mechanical resistance thus providing an ideal platform for implantation in pediatric surgery procedures such as the one for Fallot tetralogy. Amadeo et al. seeded valve interstitial cells inside decellularized porcine pericardium, to obtain a repopulated scaffold containing proliferating and mature cells capable of depositing extracellular matrix components. Given that the decellularization process removes major antigens such as αGAL (1), the Authors speculated that their procedure could be amenable to producing personalized valve tissue material suitable for re-constructing the aortic valve with no risk of rejection.

Decellularized scaffolds ideally reproduce the complex structure of the tissue of origin, however the use of natural and synthetic polymers provides a level of tunability and functionalization difficult to achieve in decellularized tissues. In particular, natural polymers are more sustainable, biocompatible and present natural ligands promoting cell adhesion. In the article collection, Majid et al.'s review summarizes the use of natural biomaterials for cardiovascular tissue engineering such as fibrinogen, collagen, alginate, silk obtained from natural sources, and Polyhydroxyalkanoates (PHAs), which can be obtained by bacterial fermentation. Alongside direct tissue replacement, biomaterials can be used to create 3D *ex vivo* models to study the processes involved in the development of congenital and acquired cardiovascular diseases, and to test novel therapeutic approaches aimed at reducing the burden of disease. The review by Iop identifies the advantages and limitations of bioengineering 3D tissue models *in vitro* for the study of common cardiovascular diseases such as arrhythmias, cardiac infections and autoimmunity, cardiovascular fibrosis, atherosclerosis, and aortic valve stenosis. A final example of cardiac disease modeling was provided in the study by Bracco Gartner et al. in which a tunable stiffness hydrogel (gelatin methacryloyl) was combined with human cardiac fibroblasts to create a 3D *in vitro* model of cardiac fibrosis. Results of this study showed the ability of these cells to modulate the mechanical properties of the extracellular matrix and thus controlling fibrosis not only through the release of paracrine signals, but also modification of tissue biophysical features.

In summary, the publications within this Research Topic illustrate the multi-faceted role of biomaterials in the fight against cardiovascular diseases, which are endemic and increasingly relevant in our society. The spectrum of the applications of these materials indicate an increasing effort toward reducing the long-term symptoms of acute cardiac events, such as valve failure or myocardial infarction, and the push to find new therapeutic options to relieve burden of congenital cardiovascular diseases.

## Author Contributions

PC and MP wrote the editorial. All authors contributed to the article and approved the submitted version.

## Conflict of Interest

The authors declare that the research was conducted in the absence of any commercial or financial relationships that could be construed as a potential conflict of interest.
